# Supplementary material for: Chromothripsis during telomere crisis is independent of NHEJ, and consistent with a replicative origin
Source: Genome Res. 2019 May;29(5):737–49. doi: 10.1101/gr.240705.118 (PMC6499312; doi:10.1101/gr.240705.118)
Supplement: Supplemental Material [file supp_gr.240705.118_Supplemental_file_1.zip › contigs/annotated_contigs/DB113/contig.2.DB113_length_600_mean_cov_12.4966666667.docx]

**DB113_length_600_mean_cov_12.4966666667**

```
AGGAAAATAAAAATACTATTTTCTGATAAGAAAATTATGTGAAATTCAAATTTCAGTGTCCCTATGTCAACTTTTATTAGAACAGCAGC
 >chr2:199687275-199687601 + E=2e-184
AGCCACATCCATTTAGGTACTGTCTGTGGCTGCTTTTACACTTCGAGTGGGATGGTAGTGACATGGAGTATGTAGCCTACCAAGCTTAA

AACATTTACTGTTTGGCCCTTTGGCTGACCTCTTGCATAAGCTATATAATCTATTTAACTCAACATGAAAGTAAGCATTCAAAGGGTTT

TCAATCCTGCAAGTAGGTGTGTCCGGAATTGGTTCCTTCCAGTGGGTTCCTGGTCTCGC|ATATATATATATGTTTTTAATTTCACATG
                                                             >chr2:199690549-199690823 +
GATTGGGTAGTTTGTAGAAAATTTCTTAGTCATATCAAAAATATGTAGAAACAATGAGCCTATAGCAGGTTAAAATGCATGCCCCATTT
 E=3e-153
GTTGATATATATTTTTGTTTAACAATCATAGAACCACCCCAACTCTCAGATGATTCTTTGTTTTTCCTTGTATAATATGCTTCACAGAC

TGATATGCAGGAGTGAGTCTAGTCAGATACCTATCCTCTGCATCTCAAAGTGGCCTTATTTCTCTTC
```
